# *Solidago canadensis* extract fractionation, phytochemical identification, and nematicidal/nematistatic activity against *Meloidogyne incognita*

**DOI:** 10.1038/s41598-026-55718-z

**Published:** 2026-06-20

**Authors:** Abdullah A. Abdel-Rahman, Shaimaa F. Diab, Shaimaa S. Shoman, Fatma S. Ahmed

**Affiliations:** 1 Zoology and Agricultural Nematology Department, Faculty of Agriculture, Cairo University, Giza, 12613 Egypt; 2 Department of Biochemistry, Faculty of Agriculture, Cairo University, Giza, 12613 Egypt; 3 Department of Economic Entomology and Pesticides, Faculty of Agriculture, Cairo University, Giza, 12613 Egypt

**Keywords:** Column chromatography, Fractions, GC‒MS analysis, Gall reduction, Nematistatic, Biological techniques, Plant sciences

## Abstract

The development of eco-friendly pest management strategies is crucial for sustainable agriculture. In this study, we investigated the bioactivity of *Solidago canadensis* leaf extract against *Meloidogyne incognita* juveniles (J2). In vitro assays showed that aqueous extracts exhibited greater nematistatic (immobilizing) activity than less polar solvent extracts. Fractionation of the aqueous decoction extract using column chromatography yielded 11 fractions (F), which were evaluated at 100, 200, and 300 mg L^− 1^. The crude extract showed 100% nematistatic activity at all concentrations, while among the fractions, F5, F7, and F9 were the most effective. In the pot experiment, the crude extract and F7 reduced gall formation by 55.3%, whereas F8, despite low in vitro nematistatic activity (21.5%), achieved the highest gall reduction (58.7%). Unexpectedly, some fractions increased gall formation. GC-MS analysis of F7 and F8 revealed 20 phytochemical compounds, including several with reported nematode-suppressive properties. These findings underscore the potential of *S. canadensis* extracts and specific fractions as botanical nematistatic agents and emphasize the importance of combining the in vitro and pot bioassays when evaluating botanical nematicides.

## Introduction

Root-knot nematodes (*Meloidogyne spp*.) are a major global agricultural threat and are responsible for significant economic losses in a wide range of crops^1^. Among them, *M. incognita* is particularly notorious for its ability to infect diverse host plants, causing root galling, impaired water and nutrient uptake, and reduced overall yield. Traditional nematode management relies heavily on synthetic nematicides, but their environmental risks, nontarget toxicity, and emergence of resistance call for alternative, effective, and sustainable strategies^2^.

Plants are promising sources of bioactive compounds with pesticidal properties, offering an eco-friendly alternative to synthetic pesticides^3–6^. Many botanicals have been introduced commercially in nematode management^4,7,8^. For example, extracts from *Acorus calamus*,* Nicotiana tabacum*,* Piper betle*,* and Syzygium* aromaticum had EC_50_ values 5–10 times lower than those of the synthetic pesticides chlorpyrifos, carbosulfan, and deltamethrin^9^.

Secondary metabolites, such as phenolic compounds, alkaloids, terpenoids, and fatty acids, play crucial roles in plant defense mechanisms^10^. These compounds exhibit broad-spectrum antimicrobial, insecticidal, and nematicidal activities, making them valuable for sustainable pest management. In the past two decades, approximately 114 plant-derived bioactive compounds from 20 plant families have been identified, including flavonoids, phenolic compounds, isothiocyanates, terpenes, alkaloids, saponins, coumarins, ketones, glucosinolates, glycosides, and limonoids. These compounds exhibit nematistatic, nematicidal, egg hatch inhibitory, membrane-disruptive, and developmental inhibitory effects against plant parasitic nematodes^4,11^.

Among the many plants studied for their phytochemical diversity and biological activities, *Solidago canadensis* (Canadian goldenrod), Asteraceae, stands out. It is a widespread perennial plant in North America, Europe, and several other countries, including China, India, Japan, Brazil, New Zealand, and Egypt^12,13^. It is also well known for its allelopathic activity and bioactive compounds, particularly flavonoids and phenolic acids^13,14^. In medicine, Solidago is a source of therapeutic compounds, such as anti-inflammatory agents^14,15^. In agriculture, it has been used for pest management; *S. canadensis* leaf essential oil (EO) has been shown to be highly toxic to *Culex quinquefasciatus* (Diptera: Culicidae), *Musca domestica* (Diptera: Muscidae), and *Spodoptera littoralis* (Lepidoptera: Noctuidae)^16^. However, limited studies have evaluated the efficacy of *S. canadensis* against plant-parasitic nematodes.

The fractionation of plant extracts is essential for identifying and isolating bioactive compounds responsible for pesticidal effects. While crude extracts provide an overview of a plant’s bioactivity, fractionation techniques such as column chromatography and solvent partitioning help pinpoint the specific components driving efficacy^17^. This approach is critical for understanding the mechanisms underlying the bioactivity, such as disruption of nematode cell membranes or inhibition of essential enzymes, and for optimizing the use of plant-derived compounds for practical applications.

To comprehensively evaluate potential bioactivity, a combination of in vitro and greenhouse experiments is necessary. In vitro assays enable precise testing of bioactivity under controlled conditions, providing primary insights into toxicity and nutritional effects on target organisms^18^. Greenhouse studies, on the other hand, bridge the gap between laboratory findings and real-world applications by assessing the efficacy of treatments in soil environments and their impact on host plants. Together, these methods provide a robust framework for evaluating the practical potential of natural products in nematode management.

Addressing this gap could provide new insights into the development of natural nematicides and contribute to sustainable agricultural practices. This study investigated the nematicidal and nematistatic effects of *S. canadensis* extracts and their fractions against *M. incognita* juveniles (J2). Additionally, the phytochemical composition of the active fractions was analyzed by gas chromatography‒mass spectrometry (GC‒MS) to identify key bioactive compounds. By integrating in vitro and greenhouse evaluations with phytochemical profiling, this research aims to advance the development of sustainable, plant-based solutions for managing root-knot nematodes and to contribute to the broader understanding of plant-derived nematicidal agents.

## Materials and methods

### Plant material and preparation

*Solidago canadensis* seedlings were obtained from Floramix Egypt Company Farms, Almansoria, Giza, Egypt (30°05′00.0″ N, 31°06′54.7″ E). The seedlings were transplanted and maintained for several months in microplots (1 m × 1 m) containing a 1:1 sandy clay: soil mixture. Fresh leaves were collected during the summer months before blooming and air-dried for one week in a shaded area. The dried leaves were coarsely ground for extraction. All experimental procedures involving plant material, including collection and preparation, were carried out in accordance with relevant institutional, national, and international guidelines and legislation. The plant was identified by members of Cairo University’s Herbarium (CAI) as *Solidago canadensis* (Asteraceae), under voucher number CAI: 105.309.10.18.

### Preparation of*S. canadensis*Extracts

Extraction was performed using three solvents with different relative polarity (R.P.): methylene chloride (R.P. = 0.309), acetonitrile (R.P. = 0.46), and water (R.P. = 1). For each extraction, 2.5 g of dried leaf powder was mixed with one liter of solvent (1X concentration). For cold-water, acetonitrile, and methylene chloride extractions, the powdered leaves were immersed in the respective solvents for 24 h at room temperature (26 ± 2 °C), then filtered through filter paper and centrifuged at 2,500 × g for 5 min to remove fine particles. For hot-water extraction, a decoction was prepared by boiling the dried leaf powder in water for 5 min on a hot plate, then cooling, filtering, and centrifuging. Organic solvents (methylene chloride and acetonitrile) were removed using a rotary evaporator, whereas water extracts were evaporated under high-speed airflow at room temperature for 24 h. The resulting crude extracts were stored at − 20 °C until further use.

### *Meloidogyne incognita* culturing

A pure culture of the root-knot nematode *M. incognita* was maintained on eggplant roots for 3 months. Roots with egg masses were incubated in a beaker of aerated water. Newly hatched juveniles (J2) were collected every 48 h via 60- and 400-mesh sieves.

### Column chromatographic analysis

The fractionation of the aqueous extract of *S. canadensis* was conducted via sequential separations via column chromatography. A column (40 cm in length and 2.5 cm in diameter) was packed with 160 g of silica gel (60–120 mesh). A total of 14.5 g of aqueous crude extract was obtained from 50 g of dry leaves. Five grams of the crude extract were ground with silica gel powder and placed on the top of a packed column. The column was eluted with different solvent mixtures, starting with 100% ethyl acetate (EA), followed by a sequential increase in polarity using ethanol (ET) and water (W) at ratios of 80:20, 60:40, 40:60, 20:80, and 0:100 (ethyl acetate: ethanol, then ethanol: water). Individual fractions of 200 mL were collected throughout the elution process. The yields were determined for EA100 (fraction 1), EA80:ET20 (F2), EA60:ET40 (F3), EA40:ET60 (F4), EA20:ET80 (F5), ET100 (F6), ET80:W20 (F7), ET60:W40 (F8), ET40:W60 (F9), ET20:W80 (F10), and W100% (F11) as 0.52 ± 0.02, 0.35 ± 0.04, 0.17 ± 0.02, 0.15 ± 0.02, 0.13 ± 0.00, 0.48 ± 0.04, 10.21 ± 0.31, 73.89 ± 4.91, 5.69 ± 0.81, 4.73 ± 0.30, 3.59 ± 0.51 mg 100 mg^− 1^ dried extract, respectively.

### GC‒MS analysis

GC‒MS/MS analysis of fractions 7 and 8 was performed via an Agilent 7000 Series Triple Quad Gas Chromatograph interfaced with a mass spectrometer (GC‒MS/MS) equipped with an Elite-5MS capillary column (5% diphenyl/95% dimethyl polysiloxane, 30 m × 0.25 μm ID × 0.25 μm df). Helium gas (99.999%) was employed as the carrier gas at a constant flow rate of 1 mL/min, and an injection volume of 2 µL was used (split ratio of 30:1). The injector temperature was set to 250 °C, and the ion-source temperature was maintained at 200 °C. The oven temperature was programmed to start at 110 °C (isothermal for 2 min), then increase at 10 °C/min to 200 °C, then at 5 °C/min to 280 °C, and finally hold at 280 °C for 9 min. Mass spectra were acquired at 70 eV, with a scan interval of 0.5 s and a mass range of 45–450 Da. The total GC running time was 36 min. The relative percentage of each component was calculated by comparing its average peak area to the total peak area. The software used to process the mass spectra and chromatograms was TurboMass. Compound identification was carried out by comparison of mass spectra with NIST and WILEY libraries, supported by diagnostic fragmentation pattern analysis according to Adams (2017)^19^, and by consistency with compounds previously reported in the literature for the same plant species^12–15,20–22^. As no authentic reference standards were analyzed, the GC–MS annotations represent putative compound identifications (Level 2) in accordance with the Metabolomics Standards Initiative (MSI) guidelines^23^.

### In vitro tests

#### First experiment

*Solidago* leaf extracts were prepared using cold water, hot water, acetonitrile, and methylene chloride. These extracts were tested at concentrations of 2500 mg L^− 1^ (1X),1250 mg L^− 1^ (0.5X), and 625 mg L^− 1^ (0.25X) to determine the most effective extract against *M. incognita* second-stage juveniles (J2). The experiment was conducted using a randomized complete block design with four independent biological replicates (*n* = 4) per treatment. Each replicate consisted of a test tube containing approximately 500 freshly hatched J2s. Tubes containing only the respective solvents were included as a control for comparison. The numbers of mobile and immobile nematodes were examined under a light microscope after 24 and 48 h of exposure. Laboratory bioassays were carried out based on the methods described by Zhang et al. (2022 with some modifications. We used 5-ml test tubes and tested a higher number of J2 (500) for each replicate, without adding any surfactants. The experiment was kept at room temperature.

#### Second experiment

The crude extracts from the hot water decoction and its fractions were tested at concentrations of 100, 200, and 300 mg L^− 1^. This experiment was repeated two times. The statistical effect was calculated as follows:

$$\:\mathrm{Nematistatic\:effect\:(\%)\:=}\frac{\mathrm{No.\:of\:immobile\:J2\:after\:48\:hr.}}{\mathrm{Total\:tested\:larvae\:after\:48\:hr.}}\mathrm{\:x\:100}$$

For each extract concentration tested, four replicates were arranged in a completely randomized design. A 0.5 mL nematode suspension (containing approximately 500 juveniles of *M. incognita*) was added to each replicate, which contained 5 mL of the extract solution in a test tube. After 48 h, 50–100 juveniles from each replicate were examined under a light binocular microscope to count mobile and immobile larvae. After examination, the nematode larvae were washed with water to remove the extraction solution, then incubated for another 48 h at room temperature. Crude extract was used as a positive control for comparison. Counts of mobile and immobile larvae were recorded again to measure the percentage of nematode larvae that regained activity after the extract was removed. Stick-like nematode larvae that did not recover after washing and showed no movement were considered dead.

### Pot experiment

The bioassay method described by Abdel-Rahman et al.^1^ was followed, with some modifications. Squash seeds were planted in 7-cm-diameter pots containing a 2:1 sand‒clay soil mixture, with one plant maintained per pot. Two weeks later, second-stage juveniles (J2) of *M. incognita* were immersed for 2 h in one of the following: Solidago crude extract as a positive control; fractions 6, 7, 8, 9, and 10; or water as a non-treated control. The immersion duration was 2 h at 300 mg L^− 1^ (the highest concentration tested in vitro) for each treatment. After immersion, the juveniles were washed with water, and each squash seedling was inoculated with approximately 800 J2. Five replicates were kept as checks, in which the inoculated nematode larvae were not treated with any extracts or fractions. The plants were maintained under horticultural conditions in a glasshouse for 40 days after inoculation. Nematode galls on the root system were then counted under a stereo microscope. Each treatment consisted of five replicates.

### Statistical analysis

To account for the non-normal distribution of proportion data and heterogeneity of variance, mortality data were analyzed using a Generalized Linear Model (GLM) with a binomial distribution and logit link function. The response variable was defined as the number of immobile juveniles (events) out of the total juveniles (trials) per replicate (*n* = 4). Analyses were conducted separately for the 24-hour and 48-hour exposure periods. Pairwise comparisons between treatments were conducted using Bonferroni-adjusted contrasts. Differences were considered statistically significant at *P* < 0.05. For the nematistatic effects, one-way ANOVAs, followed by Tukey’s HSD, were performed at each concentration (100, 200, and 300 mg L^− 1^) to compare the crude extract and all fractions. The number of galls per root and gall change relative to the control was also analyzed using one-way ANOVAs followed by Tukey’s HSD. Results are presented as mean ± standard error (SE). Statistical analyses were performed using SPSS v.22 (IBM Corp., Armonk, NY), and data visualization was carried out using GraphPad Prism v.9 (GraphPad Software, San Diego, CA, USA).

## Results

### In vitro nematicidal effects of *Solidago canadensis*

The impact of *S. canadensis* leaf extracts via different extraction methods on *M. incognita* juveniles (J2) is presented in Table 1.

**Table 1:** In vitro mortality of *M. incognita* juveniles (J2) exposed to *Solidago canadensis* leaf extracts with different extraction methods at different concentrations over 24 and 48 h.

Solvent / Treatment	Concentration	24 h Mortality (%)	48 h Mortality (%)
Aqueous Decoction ^a^ (Hot Water)	1X	96.1 ± 1.2	100.0 ± 0.0
	0.5X	96.1 ± 0.1	100.0 ± 0.0
	0.25X	95.8 ± 0.7	96.5 ± 1.9
Cold Aqueous ^b^ (Cold Water)	1X	100.0 ± 0.0	100.0 ± 0.0
	0.5X	100.0 ± 0.0	100.0 ± 0.0
	0.25X	29.5 ± 2.8	33.1 ± 1.8
Methylene Chloride ^c^ (Main effect: 24 h = c, 48 h = b)	1X	80.3 ± 4.5	100.0 ± 0.0
	0.5X	68.3 ± 1.6	100.0 ± 0.0
	0.25X	66.5 ± 2.1	100.0 ± 0.0
Acetonitrile ^d^ (Main effect: 24 h = d, 48 h = c)	1X	29.2 ± 1.8	44.9 ± 0.9
	0.5X	22.8 ± 1.2	27.6 ± 0.8
	0.25X	18.2 ± 2.6	21.0 ± 1.5
Controls			
Warm Water	-	6.41 ± 0.5	8.04 ± 0.7
Cold Water	-	8.42 ± 0.8	7.08 ± 2.0
Methylene Chloride	-	13.5 ± 1.4	18.2 ± 1.1
Acetonitrile	-	9.9 ± 0.7	14.8 ± 1.3
GLM Statistics			
Solvent Effect (χ^2^)	(df = 3)	4115.90 (*P* < 0.001)	2547.60 (*P* < 0.001)
Conc. Effect (χ^2^)	(df = 2)	1586.46 (*P* < 0.001)	1175.69 (*P* < 0.001)

Data are presented as mean ± standard error (*n* = 4). Each treatment included 4 independent biological replicates, with approximately 350–500 J2s per replicate. Superscript letters (^a, b,c, d^) indicate significant differences between solvent types (Main Effects) based on Generalized Linear Model (GLM) analysis with Bonferroni adjustment (*P* < 0.05). Values represent raw mortality percentages calculated from total counts. X = 2.5 gm dry leaves/L, 0.5 X = 1.25 gm dry leaves /L, 0.25 X = 0.6 gm dry leaves /L.

The Generalized Linear Model (GLM) analysis revealed highly significant main effects of the solvent type and concentration on *M. incognita* J2 mortality at both 24 h (χ^2^ = 4115.9, df = 3, *P* < 0.001) and 48 h (χ^2^ = 2547, df = 3, *P* < 0.001).

At 24 h, the aqueous decoction (hot water) and cold water extracts demonstrated superior efficacy, achieving near-complete mortality (96–100%) at the highest concentrations (1X and 0.5X). In contrast, the methylene chloride and acetonitrile extracts showed significantly lower activity, with acetonitrile failing to exceed 30% mortality even at the highest concentration (Table 1). Pairwise comparisons confirmed that at 24 h, all four solvent types were statistically distinct from one another (*P* < 0.001).

At 48 hours, mortality rates increased in most treatments. The methylene chloride extract showed a sharp increase in effectiveness, achieving 100% mortality across all tested concentrations and effectively ‘catching up’ to the water-based extracts. Statistical analysis revealed that although the decoction remained the most potent overall, cold water and methylene chloride had similar efficacy (*P* = 1.000), both significantly better than acetonitrile (*P* < 0.001). Control mortality was low (< 20%) across all solvent groups, confirming the bioassay’s validity.

### Nematistatic effects of the aqueous crude extract and its fractions on nematode J2

**Fig. 1:**
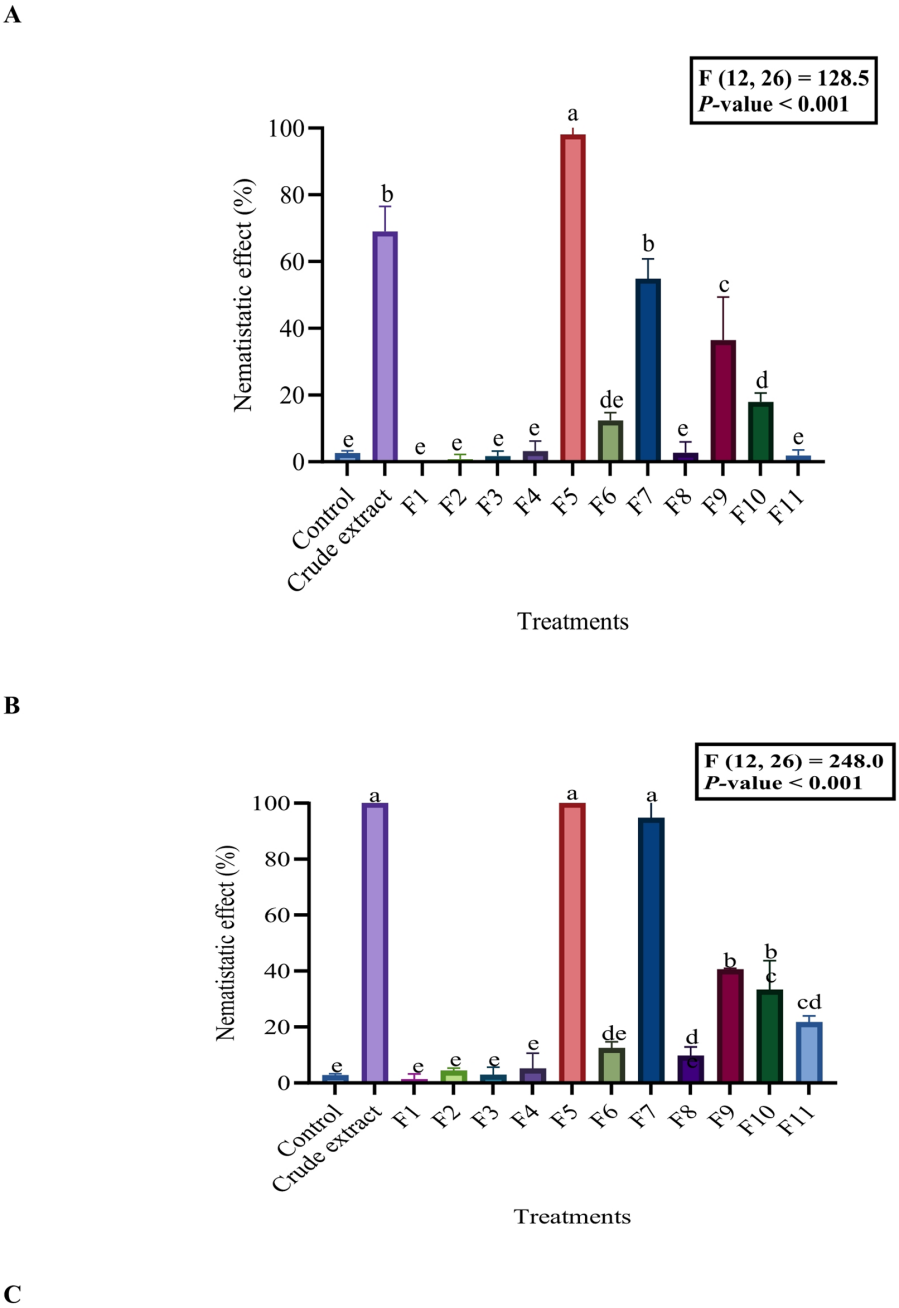

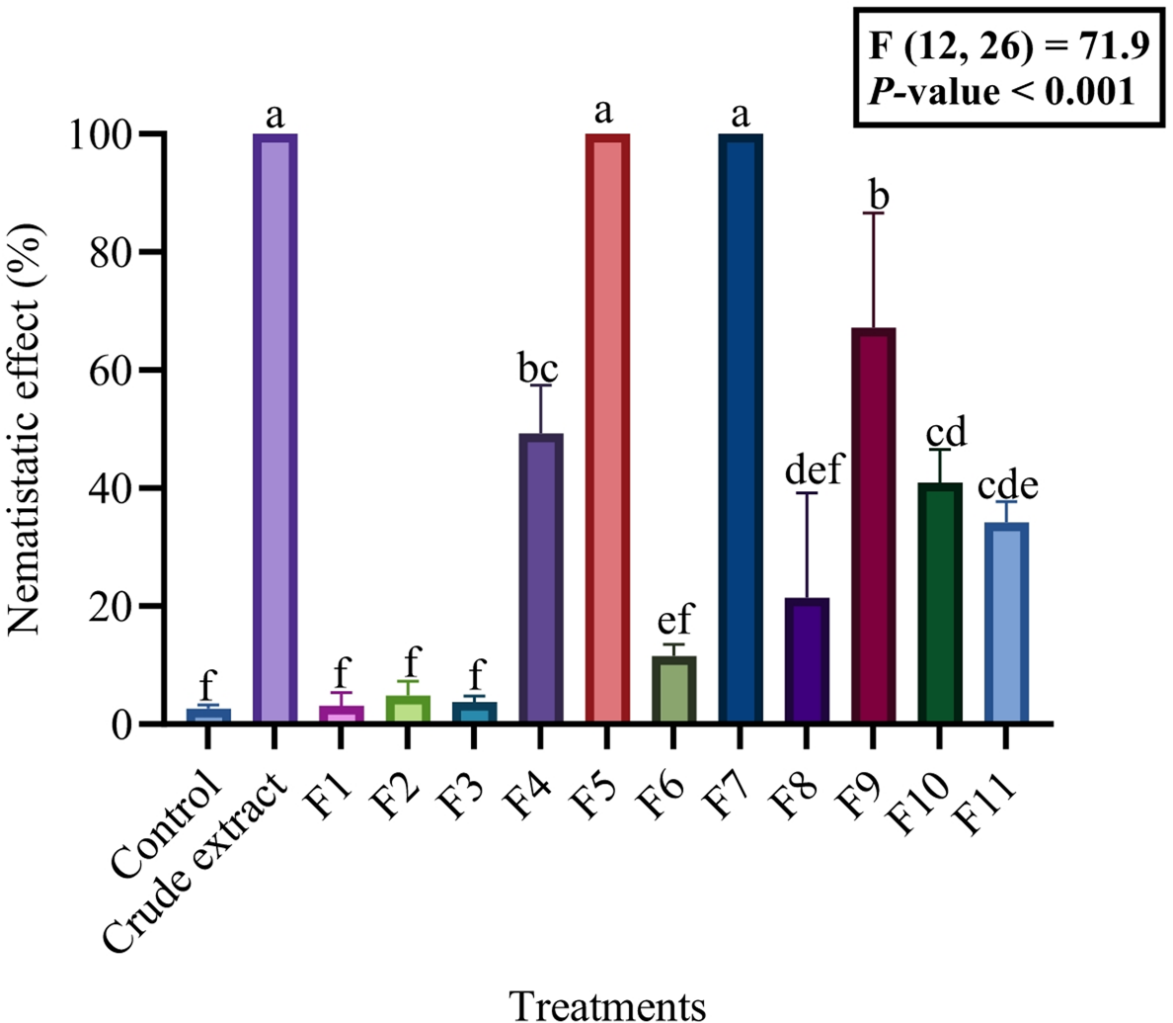
Nematistatic effects of the *Solidago canadensis* crude extract and its fractions at (A) 100 mg L^− 1^, (B) 200 mg L^− 1^, and (C) 300 mg L^− 1^ on *Meloidogyne incognita* juveniles (J2) after 48 h. Data are presented as mean ± SE; each replicate contained approximately 500 J2. Different letters above the bars indicate significant differences among treatments for each concentration level according to Tukey’s post hoc test (*P* < 0.05).

The nematistatic effects of the *S. canadensis* crude extract (C. E) and its 11 fractions (F1-F11) on *M. incognita* J2 at 3 different concentrations (100, 200, and 300 mg L^− 1^) at 48 h post-treatment are presented in Fig. 1.

The data were analyzed using one-way ANOVA separately for each concentration (100, 200, and 300 mg L^− 1^), followed by Tukey’s HSD post hoc test (*P* ≤ 0.05). Highly significant differences among treatments were observed at all concentrations (100 mg L^− 1^: F(12,26) = 128.5, *P* < 0.001; 200 mg L^− 1^: F(12,26) = 248.0, *P* < 0.001; 300 mg L^− 1^: F(12,26) = 71.9, *P* < 0.001).

#### Fractions showing significantly higher nematistatic effects

At 200 and 300 mg L^− 1^, the crude extract, Fractions EA20:ET80 (F5) and ET80:W20 (F7), were not significantly different from each other (Tukey, *P* ≤ 0.05) but were significantly higher than most other fractions. The crude extract achieved 100% immobilization at all concentrations (100, 200, and 300 mg L^− 1^ ). Fractions 5 and 7 also exhibited 100% immobilization at 200 and 300 mg L^− 1^, with F5 showing 98.1 ± 1.9% nematistatic effect at 100 mg L^− 1^. Fraction ET40:W60 (F9) had a strong nematistatic effect at 300 mg L^− 1^, with an average immobilization of 67.2 ± 11.2% (Fig. 1).

#### Fractions showing significantly lower nematistatic effects

Several fractions exhibited significantly lower nematistatic activity compared with the crude extract and the most active fractions (F5 and F7) across the tested concentrations (Tukey’s HSD, *P* ≤ 0.05). At 300 mg L^− 1^, fractions EA100 (F1), EA80:ET20 (F2), and EA60:ET40 (F3) showed very limited activity, with immobilization rates of 3.1 ± 1.3%, 4.9 ± 1.4%, and 3.8 ± 0.58%, respectively. Similarly, ET100 (F6) demonstrated low efficacy (11.5%) and remained statistically inferior to the highly active treatments (Fig. 1).

Fractions EA40:ET60 (F4) and ET20:W80 (F10) exhibited moderate but still significantly lower immobilization rates (49.3 ± 4.7% and 40.9 ± 3.2%, respectively) compared with the crude extract, F5, and F7. Fraction ET60:W40 (F8) and W100 (F11) showed intermediate activity levels (21.5 ± 5.8% and 34.3 ± 1.9%, respectively), but these values were also significantly lower than those of the most effective treatments at the same concentration (Fig. 1).

Although some fractions induced partial immobilization, their overall nematistatic performance remained statistically inferior to the crude extract and the top-performing fractions, particularly at 200 and 300 mg L^− 1^ (Fig. 1).

During the examination of the larvae, before complete immobility was observed, we noticed abnormal changes induced by certain fractions, particularly in F7, F8, F9, and F10. These abnormalities included fast repetitive stylet protraction and retraction, followed by reduced body mobility before death. In contrast, the crude extract induced abnormal hyper movement or high mobility in the larvae. The nematistatic effects of the fractions were generally very low, not exceeding 13% across all tested concentrations.

### Greenhouse experiment

Table 2 presents the efficacy of *S. canadensis* crude leaf extracts (as a positive control) and their fractions on *M. incognita* galls compared with the control. The crude extract, F7, and F8 demonstrated gall reductions of 55.3 ± 6.1%, 55.3 ± 5.5%, and 58.7 ± 2.0%, respectively, with no significant differences among them. Fraction 7 (classified as a fraction with high nematistatic effect in vitro) showed the same reduction ratio in nematode galls as the crude extract. Furthermore, F8 (classified as a fraction with low nematistatic effect in vitro) showed a 58.7% gall reduction. However, F6 showed the lowest gall reduction at 16.0%. In contrast, F9 and F10 significantly increased gall formation relative to the inoculated control, showing gall increases of 71.0% and 159.3%, respectively (*P* < 0.05).

**Table 2:** Gall reductions (%) of *Meloidogyne incognita* juveniles after exposure to *Solidago canadensis* leaf extract and its fractions.

Treatment	No. Galls/root	Gall change relative to control (%)
**Control (not inoculated)**	29.0 ± 1.2c	-
**Crude extract**	13.0 ± 3.0d	55.3 ± 6.1a
**F 6**	24.3 ± 3.2c	16.0 ± 6.5b
**F 7**	13.0 ± 2.6d	55.3 ± 5.5a
**F 8**	12.0 ± 1.0d	58.7 ± 2.0a
**F 9**	49.7 ± 6.5b	-71.0 ± 13.0c
**F 10**	75.3 ± 6.3a	-159.3 ± 12.7d
F(df)	103.5 (6, 14)	101.5(6, 14)
*P*	< 0.001	< 0.001

Data are presented as mean ± SE (*n* = 5 pots per treatment; each plant inoculated with approximately 800 J2). Different letters in the same column indicate significant differences among treatments according to Tukey’s post hoc test (*P* < 0.05). Positive values indicate a reduction in gall number compared with the inoculated control, whereas negative values indicate stimulation (increase) of gall formation.

### GC‒MS analysis of the most effective fractions against *M. incognita*

#### Phytochemical composition of (Et: Aq, 80:20), Fraction 7

GC‒MS analysis of F7 (Et: Aq 80:20) from *S. canadensis* revealed 13 phytochemicals, representing various chemical groups. The most abundant compound was the fatty acid n-decanoic acid (28.15%). Other significant components included Longifolenaldehyde (10.66%), a terpenoid. Other notable compounds included oleic acid (9.97%) and 10-heptadecen-8-ynoic acid, methyl ester (E) (9.44%). Phenolic compounds, including eugenol (8.55%) and phenol, 2-methyl-5-(1-methylethyl)- (Carvacrol) (5.53%), were highly represented (Table 3).

**Table 3:** GC–MS profile of putatively identified compounds in fraction 7 of *S. canadensis* extract.

SN	Rt	Putative compound name	Relative percentage (%)	Molecular formula	Chemical groups
1	15.64	3-Hydroxy-2-methyl-4-pyrone (Maltol)	2.94	C_6_H_6_O_3_	Aromatic and Heterocyclic Compounds
2	18.11	Benzoic acid	3.99	C_7_H_6_O_2_	Aromatic and other organic acid Compounds
3	18.93	Methyl salicylate	3.59	C_8_H_8_O_3_	Phenolic Compounds
4	20.30	Catechol	3.34	C_6_H_6_O_2_	Phenolic Compounds
5	23.35	Phenol, 2-methyl-5-(1-methylethyl)- (Carvacrol)	5.53	C_10_H_14_O	Phenolic Compounds
6	24.53	Falcarinol	3.38	C_17_H_24_O	Polyacetylene Compounds
7	24.74	Eugenol	8.55	C₁₀H₁₂O₂	Phenolic Compounds
8	29.75	n-Decanoic acid	28.15	C_10_H_20_O_2_	Fatty Acids and Lipid Derivatives
9	31.55	1,2,4-Trimethoxybenzene	4.56	C_9_H_12_O_3_	Phenolic Compounds
10	39.71	Longifolenaldehyde	10.66	C_15_H_24_O	Terpenoids
11	40.42	trans-Traumatic acid	5.90	C_12_H_20_O_4_	Aromatic and other organic acids
12	48.53	10-Heptadecen-8-ynoic acid, methyl ester, (E)-	9.44	C_18_H_30_O_2_	Fatty Acids and Lipid Derivatives
13	61.34	Oleic Acid	9.97	C_18_H_34_O_2_	Fatty Acids and Lipid Derivatives

#### Phytochemical composition of (Et: Aq, 60:40), Fraction 8

GC‒MS analysis of F8 (Et: Aq 60:40) from *S. canadensis* revealed nine phytochemicals, representing a diverse range of chemical groups. (Table 4)

**Table 4:** GC–MS profile of putatively identified compounds in fraction 8 of *S. canadensis* extract.

SN	Rt	Putative compound name	Relative percentage (%)	Molecular formula	Chemical groups
1	20.35	3-Cyclohexene-1-propanal	8.57	C_9_H_14_O	Terpenoids
2	23.32	Phenol, 2,3,5,6-tetramethyl-	19.87	C_10_H_14_O	Phenolic Compounds
3	33.92	R-Limonene	2.28	C_10_H_16_	Terpenoids
4	37.07	2,5-Octadecadiynoic acid, methyl ester	2.44	C_19_H_30_O_2_	Fatty Acids and Lipid Derivatives
5	37.77	2-Cyclohexen-1-one, 4-hydroxy-3,5,5-trimethyl-4-(3-oxo-1-butenyl)-	10.70	C_14_H_20_O_3_	Ketones (Terpenoid Derivative)
6	55.54	Carnegine	5.70	C_13_H_19_NO_2_	Alkaloids
7	58.05	1-Heptatriacotanol	50.44	C_37_H_76_O	Long-Chain Alcohols

The most abundant compound was 1-heptatriacotanol (50.44%), a long-chain alcohol. Other significant components included phenol, 2,3,5,6-tetramethyl- (19.87%), a phenolic compound.

Terpenoids included 3-cyclohexene-1-propanal (8.57%) and R-limonene (2.28%), whereas alkaloids included Carnegine (5.70%). Additional compound included 2,5-octadecadiynoic acid, methyl ester (2.44%), a fatty acid derivative.

## Discussion

The nematode mortality data highlight significant differences among extraction methods in terms of their efficacy against *M. incognita* juveniles. Aqueous decoction and cold aqueous extracts were the most effective, while the methylene chloride extract exhibited moderate efficacy. On the other hand, the acetonitrile extract demonstrated limited action against *M. incognita* larvae. Among the different fractions of the aqueous extract, increasing the ratio of polar or semipolar solvents was accompanied by higher nematistatic activity. The higher nematistatic effects come from fractions EA20:ET80 (F5), ET80:W20 (F7), and W100 (F11), while fewer nematistatic effects were associated with the EA100 fraction (F1). Considering the relative polarities of the solvents used, we suggest that polar compounds dissolved in water and in intermediate solvents contributed significantly to the higher nematistatic effects. Previous studies have revealed similar results; for instance, aqueous extracts of several medicinal plants demonstrated stronger ovicidal effects against the nematode *Hemonchus contortus* than methanolic extracts did^24^. Additionally, *Solidago* leaf water extracts exhibited greater nematistatic effects against nematodes than ethanolic extracts^25^. The nematistatic activity (31%) of the crude extract, which was absent in all other fractions, may indicate a potential synergistic interaction among the fractions.

In the pot experiment, we compared F7 and F9 (which showed high nematistatic activity, i.e., ≥ 50% at 300 mg L^− 1^) and fractions 6, 8, and 10 (which showed low nematistatic activity, i.e., < 50% at 300 mg L^− 1^) with the crude extract. Both F7 and F8 caused high gall reduction, at 58.3% and 55.3%, respectively. The in vitro nematistatic effect of F8 was low, and its high efficacy in reducing galls in the greenhouse may reflect an invisible effect on nematode larvae that is physiological in nature. Unexpectedly, both F9 (proved high nematistatic action) and F10 (low nematistatic action) increased gall formation, revealing potential nematode stimulation due to these fractions. It should be noted that no synthetic nematicide was included as a reference control in this study. The primary objective was to compare the crude extract with its derived fractions to determine whether fractionation enhanced or reduced biological activity. Therefore, the crude extract served as an internal reference for evaluating relative efficacy among fractions. Conventional nematicides typically induce 80–99% in vitro mortality depending on concentration, and previous work from our group showed that oxamyl caused 79% gall reduction in pot experiments at 250 ppm^1^. This contextual comparison helps interpret the observed gall reductions (55–58%) in the present study. One limitation of our work is that GC-MS analysis was performed only on certain fractions (F7, F8) due to their highest efficacy in the greenhouse experiments. This limited our ability to investigate why fractions 9 and 10 enhanced nematode infection.

GC‒MS results of fractions 7 and 8 identified phytochemicals that are diverse in number, ratios, and chemical groups. The composition of fraction 7 was fatty acids and lipid derivatives, terpenoids, and phenolics. Many studies have reported the antagonistic effects of various compounds against nematodes^26–29^. Regarding fatty acids, decanoic acid killed 50% of *M. incognita* juveniles at 2000 µmol L^− 1^, and it also inhibited egg hatching down to 15.8%, oleic acid also showed nematicidal activity^28^. Additionally, phenolic compounds are well known as plant defensive secondary metabolites that have suppressive bioactivities against nematodes, for instance, nematicidal and egg hatching inhibition effects^27^. Furthermore, the nematicidal activity of thirty-four terpenoids was tested; carvacrol and eugenol were among the most effective, and these effects were dose dependent^30^. The existence of such compounds in F7 as mixtures may explain its high nematicidal effect in vitro and nematode suppression in vivo.

The chromatographic analysis for F8 has detected long-chain alcohols, phenolics, terpenoids, and alkaloids. The major constituent of fraction 8, 1-heptatriacotanol, accounted for more than 50% of the fraction’s structure. A previous study on *C. elegans* concluded that alcohols can affect the worm differently depending on the carbon chain length^31^. Ethanol, a short-chain alcohol, disrupted normal movement, neural behavior, and feeding by affecting nematode neuropeptides^32,33^. Such negative effects on nematode chemotaxis and feeding suppression may not be noticeable by direct in vitro examination. This may be the reason F8 caused nematode infection failure (gall reduction = 58.7%) in the greenhouse experiment, while showing a very low nematistatic effect (21.5%) in vitro. This suggests that certain compounds in F8 may act indirectly by altering nematode sensory or behavioral responses, rather than causing immediate paralysis or mortality, or by inducing plant defense-related mechanisms in the host roots. Supporting the latter explanation, extracts of *Moringa oleifera*, *Foeniculum vulgare*, and *Chamaemelum nobile* enhanced plant defenses under nematode-induced stress^26^.

It is important to note that fraction yield does not necessarily correlate with biological efficacy. While F8 represented the highest mass recovery, its dominant constituents may possess limited direct nematicidal activity. In contrast, F7, despite its lower yield, appears enriched with bioactive compounds with stronger intrinsic potency or synergistic interactions. Therefore, the biological performance of a fraction depends more on qualitative composition and compound bioactivity than on quantitative abundance.

In fact, there is a paucity of literature regarding the bioactivity of some of the phytochemicals detected in our study. Sometimes we tried to predict their effects against nematodes using their close relatives or derivatives reported in the literature. However, differences in the phytochemical bioactivities due to simple alterations in the chemical structure were reported, even for the close derivatives of the same compound. In the alkaloids matrine, sophocarpine, and sophoramine, which share the same configuration, any structural alterations, such as the presence or absence, position, and number of double bonds, resulted in changes in nematicidal activity^26^. Additionally, oxygenated terpenoids exhibited a higher nematicidal effect than hydrocarbon terpenoids^30^. In coumarin-based compounds, carbon chain link lengths and functional moieties determined the strength of the nematicidal activity^34^. Through in vitro experiments and in silico studies, researchers provided mechanistic insights into the acetylcholinesterase-inhibitory activity of flavonoids derived from *Leucosceptrum canum*, highlighting the stronger nematicidal activity of pectolinarigenin^35^. This underscores the importance of integrating in silico and in vitro studies to elucidate the pharmacokinetics and pharmacodynamics of phytochemicals, thereby providing a clearer view of their mode of action and sustainability.

In conclusion, the findings of this study suggest that polar phytochemicals in *Solidago canadensis* leaf extract exhibit stronger nematicidal effects than nonpolar phytochemicals and that some nematistatic effects may arise from synergistic interactions among different fractions in the crude extract. Additionally, our results highlight the importance of designing a comprehensive testing system for botanical nematicide/nematistatic bioassays when evaluating natural products against nematodes, as in vitro positive or even negative effects may be altered when these products are applied in vivo. This study is limited to laboratory and greenhouse conditions. The actual field performance, long-term persistence, and environmental safety of *S. canadensis* extracts remain to be verified.

## Data Availability

The datasets generated during and/or analyzed during the current study are available from the corresponding author on reasonable request.

## Declarations

### AI use declaration

The AI-based tool (Google Gemini) was used to improve and polish the clarity and readability of an earlier version of the manuscript; no changes were made to the scientific content or conclusions. All conceptual contributions, interpretations, and scientific conclusions remain entirely the work of the authors.
